# Bacterial and Fungal Dynamics of the Uterine Microbiota in Postpartum Beef Cows Supplemented with Grape Pomace

**DOI:** 10.3390/ani16050810

**Published:** 2026-03-05

**Authors:** Inga Merkelytė, Šarūnė Marašinskienė, Rasa Nainienė, Urtė Pelenė, Laura Šakarnytė, Artūras Šiukščius

**Affiliations:** 1 Department of Animal Breeding and Reproduction, Animal Science Institute, Lithuanian University of Health Sciences, R. Zebenkos 12, 82317 Baisogala, Lithuania; sarune.marasinskiene@lsmu.lt (Š.M.); rasa.nainiene@lsmu.lt (R.N.); urte.pelene@lsmu.lt (U.P.); arturas.siukscius@lsmu.lt (A.Š.); 2 Microbiology and Virology Institute, Lithuanian University of Health Sciences, 44307 Kaunas, Lithuania; laura.sakarnyte@lsmu.lt

**Keywords:** uterus, microbiota, beef cows, postpartum, grape pomace

## Abstract

The bovine uterus, once considered sterile, is now recognized as a dynamic microbial ecosystem that undergoes substantial changes during the postpartum period and plays a critical role in reproductive health. This study investigated the composition and temporal dynamics of the uterine bacterial and fungal microbiota in postpartum Angus cows and showed that dietary grape pomace bolus supplementation was associated with temporal shifts in microbial community composition, including changes in selected bacterial taxa and increased fungal community stability. A total of 19 cows were allocated to control (*n* = 10) or treatment groups (*n* = 9), with treated animals receiving slow-release grape pomace boluses administered every three weeks from 21 days prepartum until 60–70 days postpartum. Uterine lavage samples were collected during the first (days 4–12) and ninth (days 63–70) postpartum weeks and analyzed using amplicon-based sequencing targeting the bacterial 16S rRNA (V3–V4) region and the fungal ITS2 region. The uterine microbiota exhibited pronounced temporal changes, with *Firmicutes* predominating in the early postpartum period and an increased abundance of potentially pathogenic *Fusobacteria* and *Bacteroidetes* observed by week nine postpartum, particularly in control cows. Grape pomace supplementation was associated with a more favorable bacterial profile, including a reduced relative abundance of key uterine pathogens such as *Fusobacterium necrophorum* and *Trueperella pyogenes*. The uterine mycobiome was dominated by *Ascomycota* across all groups; however, supplemented cows showed greater fungal community stability and a lower prevalence of opportunistic genera, including *Candida* spp. In conclusion, dietary grape pomace supplementation may beneficially modulate both bacterial and fungal uterine microbiota during the postpartum period, suggesting its potential as a natural strategy to support uterine microbial homeostasis and reproductive health in beef cows.

## Simple Summary

After calving, the cow’s uterus is exposed to many microorganisms that can affect recovery and future fertility. In the past, the uterus was thought to be sterile, but it is now known to contain a complex community of bacteria and fungi that changes over time. An imbalance in these microorganisms can increase the risk of uterine diseases and reduce reproductive performance. The aim of this study was to examine how the uterine microbial community changes during the weeks after calving in beef cows and to determine whether a natural dietary supplement made from grape byproducts could help maintain a healthier microbial balance. Angus cows were either given a slow-release grape pomace supplement or left unsupplemented, and uterine samples were collected early and later after calving. The results showed that harmful microorganisms became more common later after calving, especially in cows that did not receive the supplement. In contrast, cows given the grape pomace supplement showed a more stable microbial community and fewer microorganisms linked to uterine infections. These findings suggest that grape pomace, a natural and sustainable feed supplement, may help support uterine health and reduce the risk of reproductive problems in beef cows, which could improve animal welfare and productivity.

## 1. Introduction

The term microbiota refers to the diverse community of microorganisms—including bacteria, fungi, protozoa, and viruses—that inhabit specific ecological niches within a host organism [1]. Historically, the bovine reproductive tract, particularly the uterus, was considered sterile [2], but culture-independent methods, such as 16S rRNA and ITS sequencing, have revealed a resident microbiota, redefining the reproductive tract as a dynamic microbial ecosystem [2,3,4,5].

Uterine microbial composition is influenced by numerous physiological and host-related factors, including the estrous cycle phase, pregnancy status, periparturient period, parity, breed, and host genetics [2]. Among the dominant bacterial phyla reported in the healthy bovine uterus are *Bacteroidetes*, *Fusobacteria* and *Actinobacteria* [6], while other studies consistently identified four phyla—*Actinobacteria*, *Bacteroidetes*, *Firmicutes*, and *Proteobacteria*, across different uterine sites, thereby highlighting both commonalities and spatial variability [7].

Building on these observations, research has mainly focused on pathogenic bacteria in postpartum uterine diseases, as parturition represents a period of heightened susceptibility to microbial invasion [8]. The most commonly implicated pathogens include *Escherichia coli*, *Trueperella pyogenes* (formerly *Arcanobacterium pyogenes*), *Fusobacterium necrophorum*, *Bacteroides* spp., and *Prevotella* spp. [9], which, if not eliminated by the host immune system, these microorganisms can cause both subclinical and clinical infections, most notably endometritis and metritis [10].

In addition to bacteria, fungi may also play a crucial role in reproductive health. Despite extensive study of bacterial communities, fungal composition in healthy and infected cows remains largely unexplored, and emerging evidence suggests fungi may act as primary pathogens contributing to bovine infertility [11]. *Aspergillus* and *Candida* species are commonly associated with bovine abortions, primarily through direct colonization of the reproductive tract or the ingestion of mycotoxins. Although less frequent, fungal infections may also contribute to conditions such as vulvovaginitis, endometritis, and complications following intrauterine antibiotic therapy. Globally, fungal infections are estimated to account for 1–25% of all bovine abortion cases of infertility [12]. Being opportunistic in nature, fungi are more likely to establish uterine infections when predisposing conditions are present, such as prolonged intrauterine antibiotic treatment, chronic endometritis, necrotic tissue foci, immunosuppression, or pneumovagina [13].

Beyond infectious agents, both intrinsic and extrinsic factors further influence the uterine microbiota, including breed, parity, diet, housing, and peripartum events such as dystocia [1,8]. Of particular interest is the role of dietary components, such as polyphenol-rich plant supplements. Previous research has shown that grape-derived polyphenols can modulate gut microbiota [14] and may exert similar effects on reproductive tract microbial communities, reducing opportunistic taxa such as *Candida* and promoting beneficial bacteria such as *Lactobacillus* [15].

Polyphenols, especially proanthocyanidins, not only modulate microbiota but also exhibit strong antioxidant activity. An in vitro study with bovine endometrial cells demonstrated that they can significantly reduce oxidative stress caused by β-hydroxybutyrate (BHBA), a metabolite associated with ketosis and impaired reproduction in dairy cows [15]. These findings suggest that polyphenol-rich grape pomace supplementation could support uterine health during the transition period, not only through microbial modulation but also by mitigating oxidative damage to endometrial tissue—thereby potentially enhancing fertility outcomes.

The uterine microbiota also shows distinct temporal dynamics across physiological states. During pregnancy, the cervical mucus plug creates a barrier separating the uterine and vaginal microbiota [8]. In contrast, the postpartum uterus undergoes involution and is highly susceptible to microbial colonization. While the gestational uterus harbors a less diverse, but stable microbiota with minimal inflammatory response, calving disrupts this balance, increasing the risk of dysbiosis and infections caused by *Fusobacterium necrophorum*, *Trueperella pyogenes*, and *Histophilus* spp. [1].

Despite growing interest, there remains no consensus on a “core” uterine microbiota in healthy cows. Inter-study variability, due to differences in sequencing methods, sampling protocols, and geographical or genetic factors, hinders comparability [16,17]. Moreover, integrated bacterial and fungal analyses (e.g., dual 16S and ITS sequencing) remain scarce, and the temporal dynamics of the uterine microbiota across postpartum stages, particularly in beef cattle, are insufficiently characterized. Addressing these gaps could greatly enhance understanding of microbial contributions to bovine fertility and disease resilience.

The aim of this study was to investigate the composition and temporal dynamics of the uterine microbiota in Angus cows during the early and late postpartum periods, with a particular focus on differences associated with dietary supplementation of grape pomace. Specifically, the study sought to characterise changes in the uterine microbial community over time and to evaluate potential associations between grape pomace supplementation and bacterial and fungal community structure. The findings are intended to contribute to a better understanding of postpartum uterine microbial dynamics in beef cattle and their potential implications for reproductive health.

## 2. Materials and Methods

### 2.1. Animals and Experimental Design

All animal-related procedures were carried out in compliance with Directive 2010/63/EU of the European Parliament and of the Council of 22 September 2010 on the protection of animals used for scientific purposes [18]. The study also adhered to the Law of the Republic of Lithuania on Animal Welfare and Protection (Law No. IX-2271) [19], as well as to the relevant implementing regulations issued by the State Food and Veterinary Service of the Republic of Lithuania, which define the requirements for the use, housing, care, handling, and experimental application of animals [20]. Ethical approval for all experimental procedures was obtained from the Institutional Board of the Animal Science Institute, Lithuanian University of Health Sciences (protocol No. 24/01/30/01).

Nineteen Angus cows (aged 2–10 years), including both primiparous and multiparous individuals (average parity: 2.8), were enrolled in this study. All cows were clinically and reproductively healthy, as confirmed by general clinical examination and reproductive assessment, and had calved without assistance, with no reproductive complications reported before or after calving. At enrollment, all cows were in moderate body condition, with body condition scores within the recommended range for beef cattle (3.0–3.5 on a 5-point scale). The animals were assigned to one of two groups: a control group and a treatment group receiving grape pomace boluses. Multiparous cows were allocated based on parity, while primiparous cows (heifers) were randomly assigned. The control group consisted of four primiparous and six multiparous cows (average parity: 2.8), while the treatment group comprised four primiparous and five multiparous cows (average parity: 2.9).

**Figure 1:**
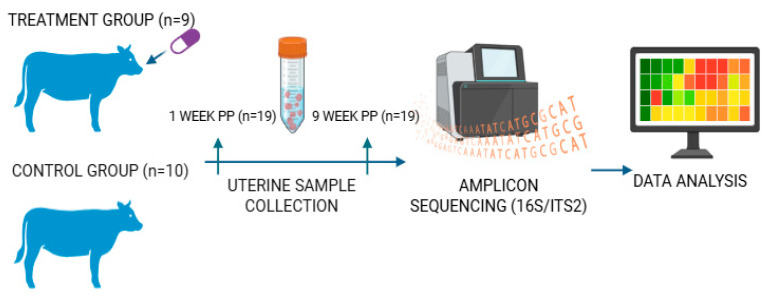
Schematic view of the study.

To assess temporal changes in the uterine microbiota, the same cows were sampled at two postpartum time points. Cows were assigned to one of two experimental groups: a treatment group receiving slow-release grape pomace boluses (*n* = 9) and a control group receiving no supplementation (*n* = 10). Uterine samples were collected from all cows during the first postpartum week (Days 4–12 postpartum) and again during the ninth postpartum week (Days 63–70 postpartum). All animals were housed in a free-stall barn located in Baisogala, Radviliškis district, in northern Lithuania. The study was conducted from spring to early summer, between March and June. From 21 days before the expected calving date until 60–70 days postpartum, cows were separated from the main herd and maintained in individual calving pens bedded with short-cut straw. Throughout the study period, both groups were fed a total mixed ration. The main components of the ration were 65% grass silage, 25% corn silage, 7% wheat straw, and 3% compound feed (corn, wheat, rape). The cows were fed once a day. In the treatment group, cows were supplemented with a slow-release grape pomace bolus containing 14.8 g of dry grape extract (*Vitis vinifera*) per bolus. The dry grape extract was encapsulated in a plant-based matrix designed to protect bioactive compounds from ruminal degradation and to allow gradual dissolution over an approximately three-week period. The bolus was characterized by a high content of polyphenols (>60%), including proanthocyanidins (>45%) and anthocyanins (>0.3%). Boluses were administered every three weeks starting 21 days before calving and continued until 60–70 days postpartum, ensuring uninterrupted exposure to bioactive compounds throughout the supplementation period. A schematic representation of the study is presented in Figure 1.

### 2.2. Uterine Sample Collection

Before uterine sampling, the perineum of each restrained cow was thoroughly cleaned with water and disinfected with 70% ethanol, followed by drying with a clean paper towel. A sterile plastic infusion pipette was gently introduced through the vagina and cervix into the uterine body by an experienced technician. Ultrasound guidance was used as an additional precaution to assist in visualization of the reproductive tract and to confirm correct catheter placement. Ultrasonography was performed using an Easi-Scan Go ultrasonic scanner equipped with a 7.5 MHz linear rectal transducer (BCF™ Technology Ltd., Livingston, UK).

Subsequently, 30 mL of sterile saline solution was infused into the uterus. The uterus was gently massaged externally via rectal palpation to mix the solution with uterine contents, after which the lavage fluid was aspirated and recovered. The recovered volume ranged between 5 and 15 mL. Uterine samples were immediately transferred into pre-labeled sterile 15 mL conical tubes, placed in a Styrofoam container with ice, and transported to the laboratory within 2–3 h for further processing. Equal volumes from individual samples were pooled into representative composite samples and stored in sterile cryogenic tubes at −80 °C at the Microbiology and Virology Institute, Lithuanian University of Health Sciences. Samples were subsequently transported on ice in DNA/RNA Shield (Zymo Research, Irvine, CA, USA) to the designated sequencing facility. To enable a cost-effective initial characterization of the uterine microbiome, samples were pooled prior to sequencing. Although this approach limits individual-level resolution, it allowed exploratory analysis of overall microbial community composition in beef cows and provided baseline data for future, more detailed investigations.

### 2.3. Amplicon-Based Sequencing of Bacterial (16S) and Fungal (ITS2) Communities

DNA extraction, library preparation, and targeted amplicon sequencing were conducted using the ZymoBIOMICS^®^ Targeted Sequencing Service (Zymo Research, Irvine, CA, USA). Genomic DNA was extracted from uterine lavage samples using the ZymoBIOMICS^®^ DNA Miniprep Kit.

For bacterial community profiling, the V3–V4 region of the 16S rRNA gene was PCR-amplified using the primer set included in the Quick-16S™ NGS Library Prep Kit (Zymo Research, Irvine, CA, USA). For fungal community profiling, the ITS2 region was PCR-amplified using the Quick-ITS™ Plus NGS Library Prep Kit with the Microbiota Sequencing Services ITS2 Primer Set (Zymo Research, Irvine, CA, USA). Library preparation: PCR was performed on real-time PCR platforms to control amplification cycles and minimize chimera formation. PCR products were quantified by qPCR, pooled equimolarly, purified with the Select-a-Size DNA Clean & Concentrator™ kit (Zymo Research, Irvine, CA, USA), and quantified using TapeStation^®^ (Agilent Technologies, Santa Clara, CA, USA) and Qubit^®^ (Thermo Fisher Scientific, Waltham, MA, USA).

Positive controls included the ZymoBIOMICS^®^ Microbial Community Standard (for DNA extraction) and the ZymoBIOMICS^®^ Microbial Community DNA Standard (for library preparation). Negative controls (blank extractions and library preparations) were processed in parallel to assess potential contamination.

Bacterial libraries were sequenced on the Illumina^®^ NextSeq™ platform (P1 reagent kit (Illumina Inc., San Diego, CA, USA), (600 cycles, 30% PhiX spike-in), while fungal libraries were sequenced on the Illumina^®^ NextSeq 2000™ platform (P1 reagent kit (Illumina Inc., San Diego, CA, USA), (600 cycles, 25% PhiX spike-in).

### 2.4. Bioinformatics and Quantification

Raw sequence reads were trimmed for quality and adapter removal. Unique amplicon sequence variants (ASVs) were inferred using the DADA2 pipeline [21], and chimeras were removed. Taxonomic assignment was performed with Uclust in QIIME v.1.9.1 [22] using the Zymo Research 16S and ITS Databases (curated internal reference databases). Microbial community composition was analyzed in QIIME v.1.9.1. Differentially abundant taxa were identified using LEfSe v1.1.2 [23] with default parameters, while heatmaps, Taxa2ASV/Taxa2SV decomposer, and PCoA plots were generated to visualize overall community composition using in-house scripts.

Absolute abundance was assessed by quantitative PCR (qPCR) using plasmid DNA standards containing one copy each of the 16S and ITS2 regions prepared in 10-fold serial dilutions. Gene copy numbers were converted to genome copies, assuming four 16S copies per bacterial genome and 200 ITS copies per fungal genome. DNA mass was estimated based on genome sizes of 4.64 × 10^6^ bp for *Escherichia coli* (16S) and 1.20 × 10^7^ bp for *Saccharomyces cerevisiae* (ITS2). Sequences were deposited in the NCBI database under accession number PRJNA1321294.

## 3. Results

### 3.1. Microbial Composition of Uterine Microbiotas of Beef Cows

**Figure 2:**
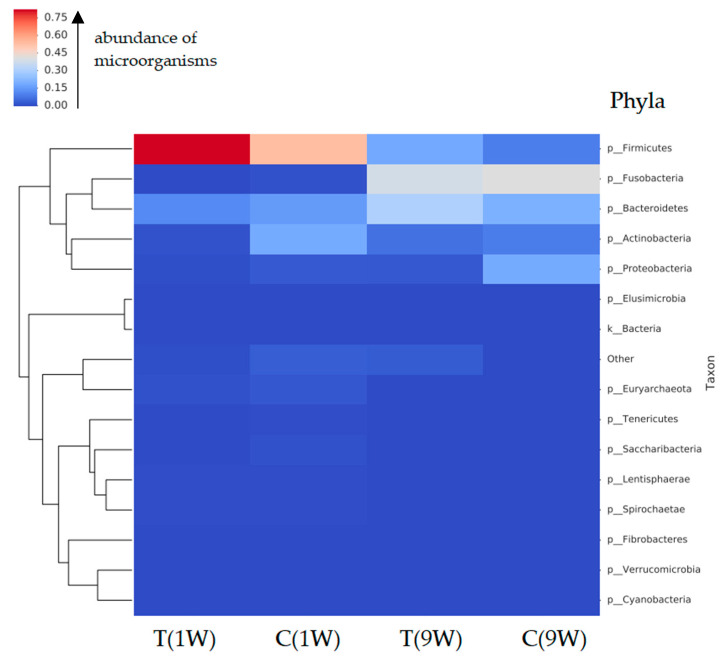

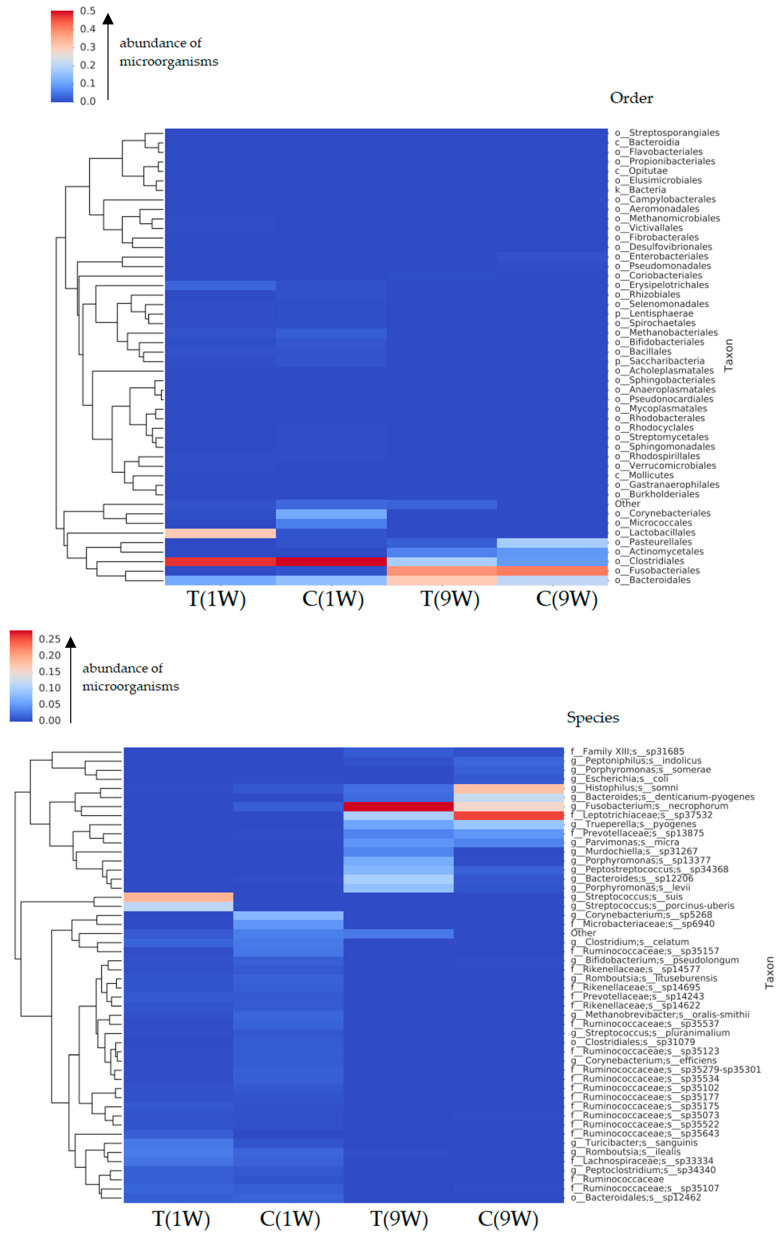
Taxonomic composition of the uterine microbiota in beef cows according to treatment and postpartum sampling time. The treatment group was sampled at 1 week (T(1w)) and 9 weeks (T(9w)) postpartum, and the control group was sampled at the same time points (C(1w) and C(9w)). The microbiota is presented at the phylum, order, and species levels. The color scale represents relative abundance, ranging from dark red (highest abundance) to dark blue (lowest abundance). Each row corresponds to a specific taxon, with taxonomic identifiers shown on the right. Samples were hierarchically clustered using Bray–Curtis dissimilarity. Taxon names ending with alphabetic suffixes indicate polyphyletic groups.

Figure 2 illustrates the bacterial composition of the uterine microbiota of beef cows at the phylum, order, and species levels.

Data from Figure 2 revealed a distinct distribution of bacteria at the phylum level across all four sampling categories defined by treatment and postpartum time. The uterine microbiota of cows sampled at 1 week postpartum, both in the treatment (T (1w)) and control (C (1w)) groups, was dominated by *Firmicutes*, whereas samples collected at 9 weeks postpartum from both the treatment (T (9w)) and control (C (9w)) groups were dominated by *Fusobacteria*. Differences in bacterial composition were also evident at the order level. *Clostridiales* and *Lactobacillales* were prevalent in the treatment group at 1 week postpartum (T (1w)), while *Clostridiales* predominated in the control group at the same time point (C (1w)). In contrast, *Fusobacteriales* and *Bacteroidales* were most frequently detected in the treatment group at 9 weeks postpartum (T (9w)), whereas *Fusobacteriales* dominated in the control group at 9 weeks postpartum (C (9w)).

To further characterize differences in microbiota composition, taxonomic analysis was extended to the species level. *Streptococcus* species predominated in the treatment group sampled at 1 week postpartum (T (1w)), while *Corynebacterium* was the most abundant genus in the corresponding control group (C (1w)). At 9 weeks postpartum, the uterine microbiota of the treatment group (T (9w)) was dominated by *Fusobacterium* species, whereas samples from the control group (C (9w)) showed a higher relative abundance of *Leptotrichiaceae*, *Histophilus*, and *Fusobacterium* taxa.

### 3.2. Analysis of Bacterial Phyla–Structure and Significance in the Bovine Microbiota

**Figure 3:**
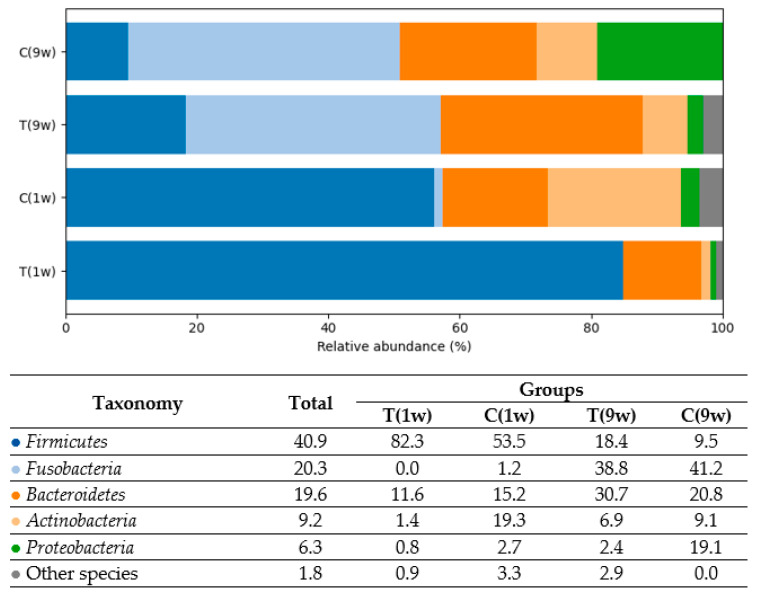
Comparison of bacterial genera composition in the uterine microbiota of beef cows across experimental groups. T(1w)—treatment group at 1 week postpartum; C(1w)—control group at 1 week postpartum; T(9w)—treatment group at 9 weeks postpartum; C(9w)—control group at 9 weeks postpartum. Values represent the relative abundance (%) of individual bacterial genera.

Figure 3 illustrates the genus-level microbial composition of the uterine microbiotas across four groups of beef cows.

Only genera with a relative abundance ≥1% in at least one group are presented. Values in the table represent the percentage abundance of each genus.

**Figure 4:**
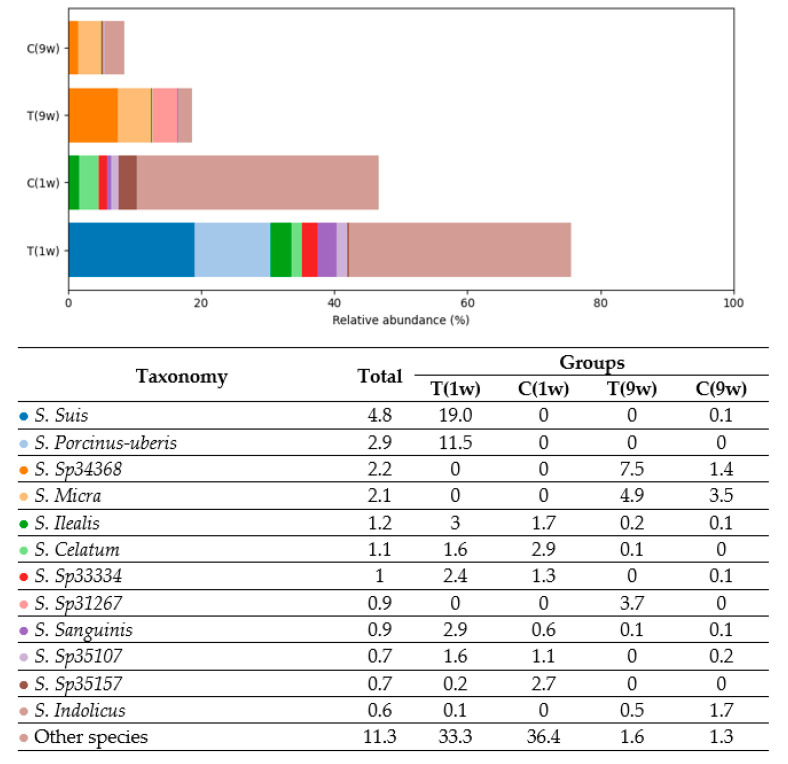
Comparison of the relative abundance of *Firmicutes* species (% of total bacterial composition) in beef cows. T(1w)—treatment group at 1 week postpartum; C(1w)—control group at 1 week postpartum; T(9w)—treatment group at 9 weeks postpartum; C(9w)—control group at 9 weeks postpartum.

**Figure 5:**
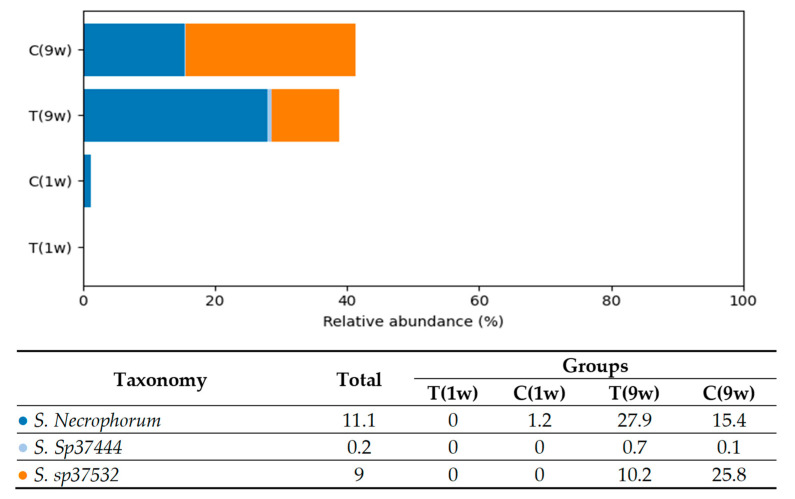
Comparison of the relative abundance of *Fusobacteria* species (% of total bacterial composition) in beef cows. T(1w)—treatment group at 1 week postpartum; C(1w)—control group at 1 week postpartum; T(9w)—treatment group at 9 weeks postpartum; C(9w)—control group at 9 weeks postpartum.

**Figure 6:**
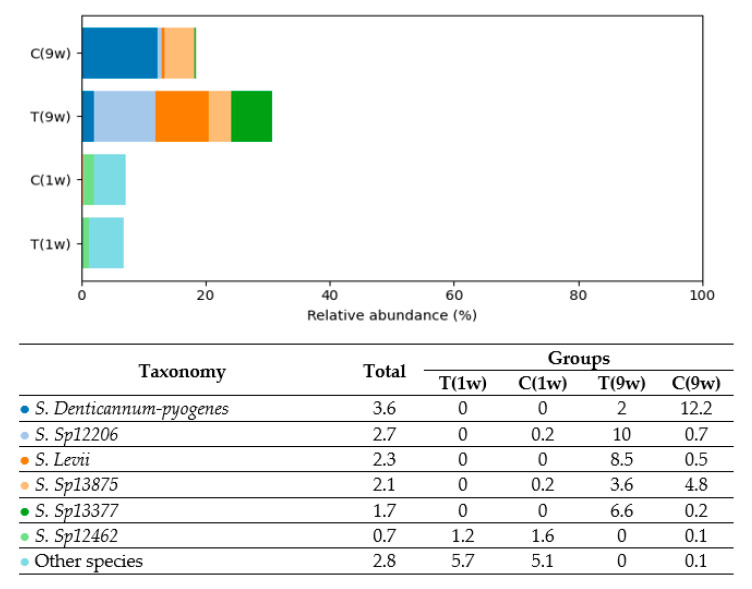
Comparison of the relative abundance of *Bacteroidetes* species (% of total bacterial composition) in beef cows. T(1w)—treatment group at 1 week postpartum; C(1w)—control group at 1 week postpartum; T(9w)—treatment group at 9 weeks postpartum; C(9w)—control group at 9 weeks postpartum.

**Figure 7:**
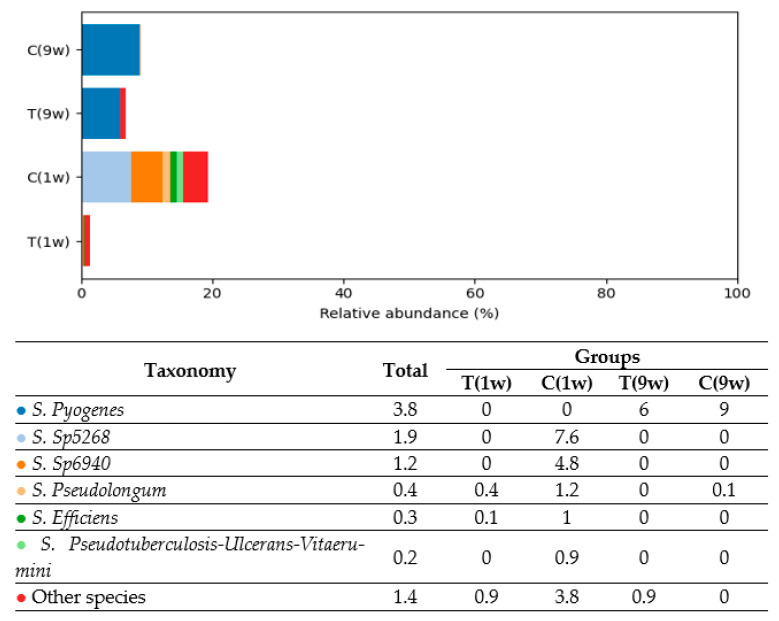
Comparison of the relative abundance of *Actinobacteria* species (% of total bacterial composition) in beef cows. T(1w)—treatment group at 1 week postpartum; C(1w)—control group at 1 week postpartum; T(9w)—treatment group at 9 weeks postpartum; C(9w)—control group at 9 weeks postpartum.

**Figure 8:**
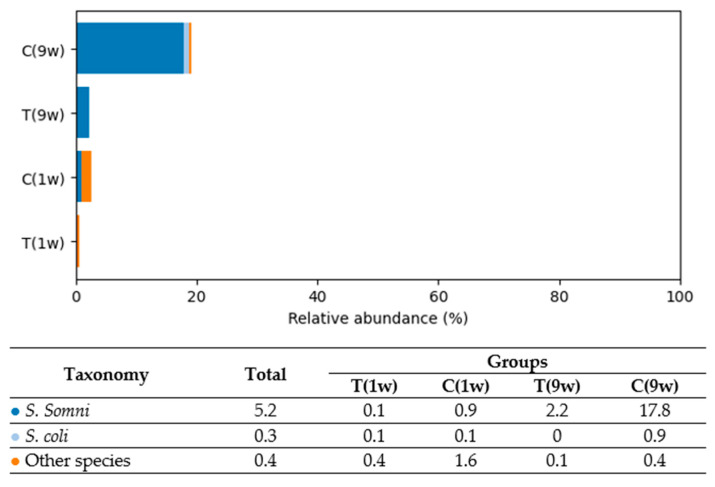
Comparison of the relative abundance of Proteobacteria species (% of total bacterial composition) in beef cows. T(1w)—treatment group at 1 week postpartum; C(1w)—control group at 1 week postpartum; T(9w)—treatment group at 9 weeks postpartum; C(9w)—control group at 9 weeks postpartum.

Based on the analysis, five predominant bacterial phyla were identified in the uterine microbiota of beef cows: *Firmicutes* (40.9%), *Fusobacteria* (20.3%), *Bacteroidetes* (19.6%), *Actinobacteria* (9.2%), and *Proteobacteria* (6.3%). In total, 605 bacterial species were detected in the uterine microbiota, reflecting a high level of taxonomic diversity across all experimental groups. Figure 4, Figure 5, Figure 6, Figure 7 and Figure 8 illustrate the dominant species within the most prevalent genera, highlighting their taxonomic classification, potential functional roles, and distribution across experimental groups.

Analysis of *Firmicutes* species revealed distinct compositional differences across cow groups (see Figure 4). In Group T(1w), the microbiota was dominated by *Streptococcus Suis* and *Streptococcus porcinus-uberis*, comprising 19.0% and 11.5% of the total bacterial community, respectively. These species were nearly absent in the remaining groups.

In Group C(1w), the most abundant *Firmicutes* species included *Clostridium celatum* (2.9%), *Lactobacillus ilealis* (1.7%), and several unidentified *Clostridiales* species, such as *Sp33334* and *Sp35107*. In contrast, Groups T(9w) and C(9w) exhibited significantly lower overall abundance of *Firmicutes* species, with only trace levels (0.1–0.2%) of *L. ilealis*, *C. sanguinis*, and other taxa detected. These findings demonstrate clear taxonomic divergence among groups: Group T(1w) was dominated by lactic acid bacteria (*Streptococcus* spp.), Group C(1w) by intestinal anaerobes (*Clostridium*, *Lactobacillus*), while Groups T(9w) and C(9w) displayed markedly reduced representation of *Firmicutes* taxa.

Analysis of *Fusobacteria* species revealed a heterogeneous distribution among experimental groups (see Figure 5). In Groups T(9w) and C(9w), *Fusobacterium necrophorum* emerged as the dominant species, accounting for 27.9% and 15.4% of the total microbiota, respectively. This species is a well-established pathogen in cattle and is frequently implicated in necrotic conditions such as metritis and hepatic abscesses, and it is also naturally found in the bovine gastrointestinal and reproductive tracts. Another highly abundant species, *Fusobacterium sp37532*, represented 10.2% of the microbiota in Group T(9w) and 25.8% in Group C(9w). Although its taxonomy remains incomplete, the prevalence of this species further emphasizes the dominance of *Fusobacteria* in these two groups. In contrast, *Fusobacterium sp37444* was detected at only low levels (up to 0.7%) in Group T(9w), and overall *Fusobacteria* abundance was negligible in Groups T(1w) and C(1w).

Analysis of uterine microbiota composition revealed distinct group-specific patterns in *Bacteroidetes* distribution (see Figure 6). These taxa were detected exclusively in Groups T(9w) and C(9w), while remaining absent or below detection limits in Groups T(1w) and C(1w). In Group C(9w), *Bacteroides denticanum-pyogenes* was the dominant taxon, comprising 12.2% of the total microbiota—the highest relative abundance of any *Bacteroidetes* species across all groups. Additional taxa detected in this group included *Sp13875* (4.8%), *Sp12206* (0.7%), and *Bacteroides levii* (0.5%), indicating higher species-level diversity within the phylum.

Group T(9w) exhibited a moderately diverse *Bacteroidetes* profile, with *Sp12206* accounting for 10.0%, *B. levii* for 8.5%, and *Sp13875* for 3.6% of the microbial community. Although present in lower proportions than in Group C(9w), the range of species still reflects notable diversity. In contrast, *Bacteroidetes* taxa were not detected in Groups T(1w) and C(1w), suggesting their absence or occurrence at levels below the threshold of detection in those animals.

Analysis of *Actinobacteria* composition revealed group-specific dominance by distinct taxa (see Figure 7). In Group C(1w), the most prevalent sequences included two unidentified species—*Sp5268* (7.6%) and *Sp6940* (4.8%)—alongside *Bifidobacterium pseudolongum* (1.2%) and *B. Efficiens* (1.0%). These findings suggest a more diverse and pronounced *Actinobacteria* community in this group. Conversely, Groups T(9w) and C(9w) were predominantly characterized by the presence of *Trueperella pyogenes*, accounting for 6.0% and 9.0% of the microbiota, respectively. This species is a well-known opportunistic pathogen frequently associated with uterine and respiratory infections in cattle. In Group T(1w), *Actinobacteria* were detected at minimal levels, with only trace amounts of *S. pseudolongum* (0.4%) and *S. efficiens* (0.1%) observed.

Analysis of *Proteobacteria* distribution (see Figure 8) revealed the highest abundance of this phylum in Group C(9w). The dominant species was *Histophilus somni*, which accounted for 17.8% of the total microbiota in this group. In comparison, *H. somni* was present at lower levels in Group T(9w) (2.2%), Group C(1w) (0.9%), and Group T(1w) (0.1%). Another detected species, *Escherichia coli*, was found in low abundance across all groups, ranging from 0.0% to 0.9%.

### 3.3. Fungal Composition of the Uterine Microbiota in Beef Cows

**Figure 9:**
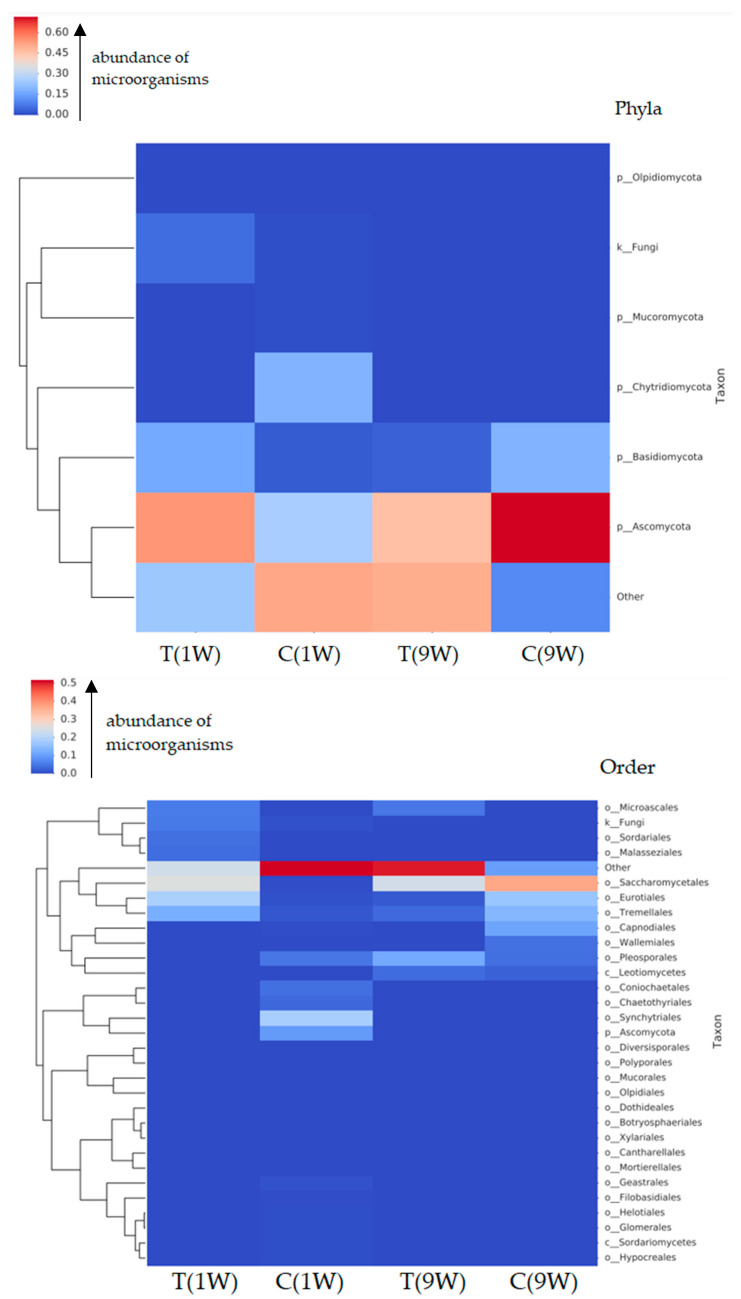

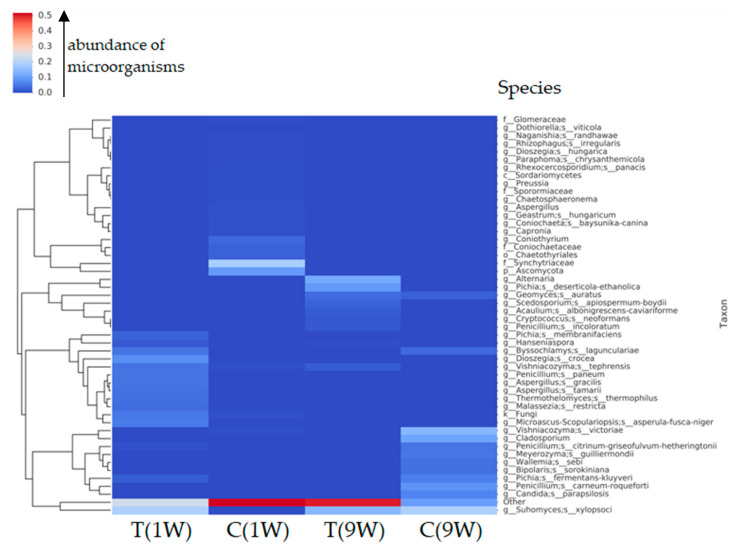
Composition of the uterine microbiota in beef cows according to treatment group and postpartum sampling time (T(1w), C(1w), T(9w), and C(9w)). The microbiota is presented at the phylum, order, and species levels. Color intensity indicates relative abundance, ranging from dark red (highest) to dark blue (lowest). Each row represents a taxon, with taxonomic identifiers shown on the right. Samples were hierarchically clustered using Bray–Curtis dissimilarity. Taxon names ending with alphabetic suffixes indicate polyphyletic groups.

Fungal compositions at the phylum, order, and species levels of uterine microbiotas of beef cows are presented in Figure 9.

The heatmap of fungal taxa at the order level revealed clear differences in fungal community composition across cow groups. Notably, *Saccharomycetales* was the most dominant fungal order, particularly in Group C(1w), where it displayed the highest relative abundance (deep red), followed by moderate representation in Group T(9w) and minimal presence in Group C(9w). This group-specific increase indicates that, in the uterine environment of Group C(1w) animals, these fungi may have proliferated more actively or become more firmly established. Group T(1w) exhibited a relatively low abundance across most taxa, with slight signals from *Microascales*, *Sordariales*, and *Eurotiales*. In contrast, Group C(9w) displayed generally low richness and evenness at the order level, lacking strong representation of any specific order.

Other notable orders included *Eurotiales*, *Tremellales*, and *Sordariales*, which showed moderate to low abundances in Groups T(1w) and T(9w). *Malasseziales* and *Capnodiales* were also observed, but at lower frequencies, indicating a secondary role in the overall community structure.

The “Other” category showed varying levels across groups, suggesting the presence of less-characterized or low-abundance taxa that may still contribute to functional dynamics within the uterine mycobiome.

The heatmap analysis of fungal taxa at the phylum level revealed distinct compositional differences among the experimental groups. In total, 83 phyla were identified, several of which showed marked variation in relative abundance across groups. The most dominant phylum across all groups was *Ascomycota*, showing highest relative abundance in Group C(9w), followed by Groups T(9w), C(1w), and T(1w). Notably, Group C(9w) exhibited the most intense signal (dark red), indicating a pronounced *Ascomycota* dominance in this group.

*Basidiomycota* was also present in all groups, with slightly higher abundance in Group C(9w) compared to the others, although its overall representation remained lower than that of *Ascomycota.*

*Chytridiomycota* and *Mucoromycota* were observed primarily in Groups C(1w) and T(9w), suggesting possible group-specific colonization or environmental exposure. *Olpidomycota* and unclassified fungal sequences (e.g., k. Fungi) showed minimal representation, limited to Group T(1w).

The “Other” category, comprising low-abundance or poorly classified taxa, was most pronounced in Groups 2 and 3, indicating higher overall.

These findings suggest that *Ascomycota* is the core fungal phylum in the bovine uterus, while other phyla such as *Basidiomycota*, *Chytridiomycota*, and *Mucoromycota* contribute to group-specific variation, potentially influenced by physiological, environmental, or treatment-related factors.

Species-level analysis of the uterine fungal microbiota revealed highly differentiated profiles among the cow groups. The genus *Suhomyces*, particularly *S. xylopsoci*, was the most dominant species, showing the highest relative abundance in Groups C(1w) and T(9w), with notable presence in Group T(1w) and minimal representation in Group C(9w). This consistent dominance suggests that *S. Xylopsoci* may serve as a core species within the uterine mycobiome of certain individuals.

Group T(1w) showed a moderate variety of species, including *Penicillium paneum*, *Aspergillus tamarii*, and *Vishniacozyma tephrensis*, indicating a stable fungal environment likely influenced by endogenous microbial dynamics. Group C(1w), in contrast, showed increased richness in species such as *Pichia deserticola-ethanolica*, *Geomyces Auratus*, and *Scopulariopsis apiospermum-boydii*, suggesting possible environmental colonization or post-calving microbial expansion.

Group T(9w) exhibited a diverse but transitional profile, characterized by moderate levels of *Penicillium citrinum-griseofulvum-hetheringtonii*, *Candida parapsilosis*, and *Pichia fermentans-kluyveri*, possibly indicating a shift toward opportunistic fungal colonization. Group C(9w), by contrast, showed relatively low species diversity and abundance, with the exception of *Suhomyces xylopsoci*, which remained detectable at lower levels.

Interestingly, several potentially pathogenic or environmental fungi—including *Cryptococcus neoformans*, *Cladosporium*, *Malassezia restricta*, and *Aspergillus gracilis*—were sporadically present across groups, though not dominant. Their low-level presence may reflect transient colonization or suppressed growth under specific uterine conditions.

Overall, the species-level distribution patterns reflect both stable and dynamic aspects of the bovine uterine mycobiome, influenced by group-specific factors such as immune status or metabolic conditions.

**Figure 10:**
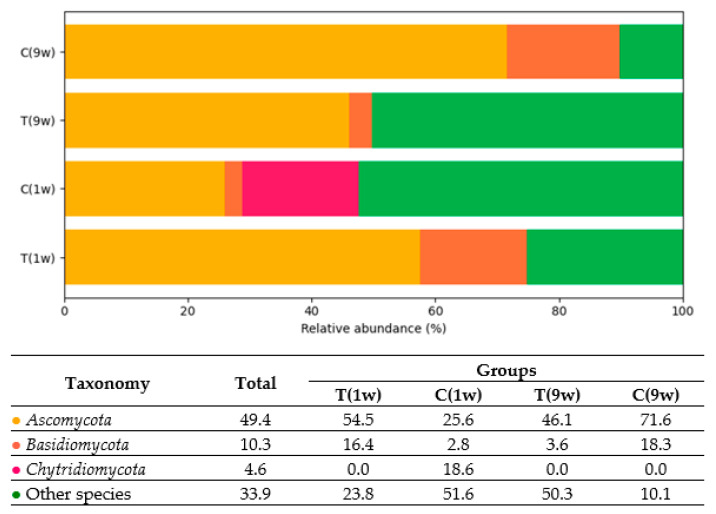
Comparison of fungal genera of uterine microbiotas of beef cows. Only genera with a relative abundance ≥ 1% in at least one group are presented. Values in the table represent the percentage abundance of each genus. T(1w)—treatment group at 1 week postpartum; C(1w)—control group at 1 week postpartum; T(9w)—treatment group at 9 weeks postpartum; C(9w)—control group at 9 weeks postpartum.

Based on the presented data, three predominant fungi were identified in the uterine microbiota of beef cows: *Ascomycota* (49.4%), *Basidiomycota* (10.3%), and *Chytridiomycota* (4.6%) as shown in Figure 10.

**Figure 11:**
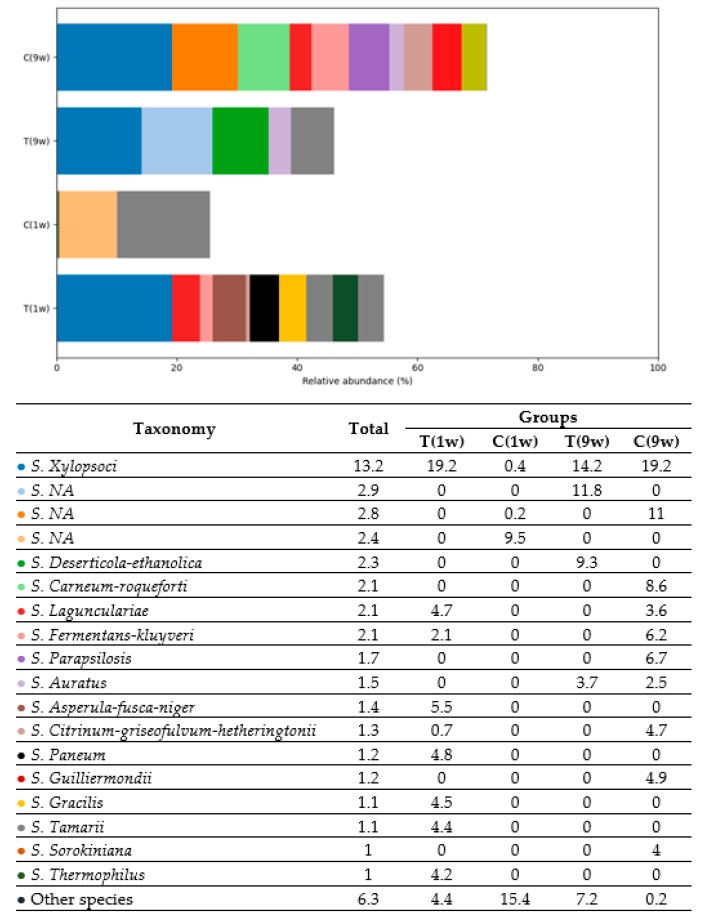
Relative abundance of *Ascomycota* species (% of total fungal composition) in beef cows. T(1w)—treatment group at 1 week postpartum; C(1w)—control group at 1 week postpartum; T(9w)—treatment group at 9 weeks postpartum; C(9w)—control group at 9 weeks postpartum.

**Figure 12:**
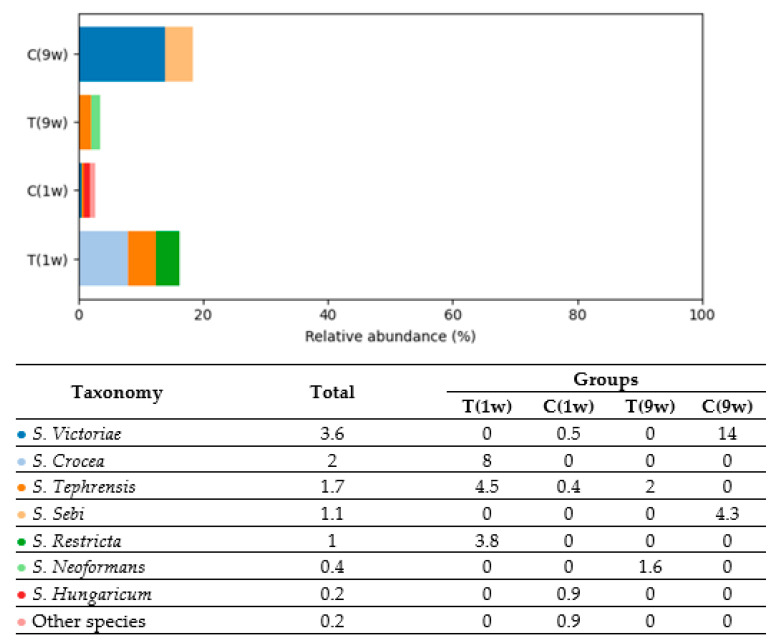
Relative abundance of *Basidiomycota* species (% of total fungal composition) in beef cows. T(1w)—treatment group at 1 week postpartum; C(1w)—control group at 1 week postpartum; T(9w)—treatment group at 9 weeks postpartum; C(9w)—control group at 9 weeks postpartum.

Fungal species of the most prevalent genera detected in beef cows are presented in Figure 11 and Figure 12.

In Group T(1w), the dominant taxon was *S. Xylopsoci* (19.2%), followed by *S. Asperula-fusca-niger* (5.5%), *S. Paneum* (4.8%), *S. Gracilis* (4.5%), *S. Tamarii* (4.4%), and *S. Thermophilus* (4.2%). This group displayed high *Ascomycota* diversity, with multiple species from genera such as *Aspergillus* and *Penicillium*. Group C(1w) exhibited a more limited profile, dominated by *S. Deserticola-ethanolica* (9.3%) and *NA* (9.5%), along with trace levels of *S. Carneum-roqueforti* (8.6%) and *S. Fermentans-kluyveri* (6.2%). The presence of multiple unidentified taxa suggests lower classification resolution in this group. In Group T(9w), *S. Xylopsoci* remained the dominant species (14.2%), accompanied by *S. Apiospermum-boydii* (3.0%), *S. Albonigrescens-caviariforme* (1.8%), *S. Incoloratum* (1.6%), and several low-abundance *NA* entries. This composition suggests a transitional fungal profile, possibly reflecting environmental exposure or early microbial colonization shifts. Group C(9w) exhibited the highest fungal diversity, with co-dominance of *S. Xylopsoci* (19.2%), *NA* (11.0%), *S. Guilliermondii* (4.9%), *S. Parapsilosis* (6.7%), *S. Citrinum-griseofulvum-hetheringtonii* (4.7%), and *S. Membranifaciens* (2.7%). The substantial presence of both classified and unclassified fungi suggests a highly complex and dynamic fungal environment. Such diversity may reflect the influence of multiple physiological factors, including the groups’ immune status, tissue microenvironment, and overall health condition, which can shape the composition and activity of fungal communities.

Group C(9w) was dominated by *S. Victoriae*, which accounted for 14.0% of the total fungal community—its highest observed abundance among all groups. *S. Sebi* also appeared exclusively in this group, at a relative abundance of 4.3%, suggesting a unique fungal composition compared to other groups. In Group T(1w), the predominant species were *S. Crocea* (8.0%), *S. Tephrensis* (4.5%), and *S. Restricta* (3.8%). This group exhibited a moderately diverse fungal profile composed primarily of environmental molds.

Group T(9w) showed lower diversity but a notable presence of *S. Tephrensis* (2.0%) and *S. Neoformans* (1.6%), the latter being a potentially pathogenic yeast commonly found in environmental reservoirs and occasionally associated with host infection. In Group C(1w), only low-abundance species were detected, such as *S. Victoriae* (0.5%), *S. Tephrensis* (0.4%), and *S. Hungaricum* (0.9%), indicating limited fungal colonization or presence below detectable thresholds in most taxa.

## 4. Discussion

One of the key challenges in modern cattle production is to understand postpartum uterine microbial dynamics while minimizing the risk of uterine infections that may impair reproductive efficiency. Following calving, the uterus is inevitably exposed to environmental and endogenous microorganisms, and microbial succession during this period is considered a normal component of uterine involution [24]. Previous studies have demonstrated that the bovine postpartum uterus is rapidly colonized by diverse microbial communities and that their composition undergoes marked temporal changes during the early postpartum period [7,24].

In our study, phylum-level analysis revealed five dominant bacterial phyla across all experimental groups: *Firmicutes*, *Fusobacteria*, *Bacteroidetes*, *Actinobacteria*, and *Proteobacteria*. This composition is consistent with previous reports describing the core postpartum uterine microbiota in cattle [7,24,25,26]. Several additional low-abundance phyla, including *Tenericutes, Spirochaetae*, and *Verrucomicrobia*, were also detected, reflecting the high microbial diversity typically observed in the postpartum uterus. Temporal changes in uterine microbial composition observed in our study are consistent with findings reported by other investigators. Knudsen et al. (2016) [27] reported temporal variation in the postpartum uterine microbiota, showing that bacterial community structure differs between early and later postpartum stages and between clinically healthy and diseased cows. Similarly, Pascottini et al. (2020) [16] reported marked time-dependent shifts in the uterine microbiota, which they interpreted as part of physiological uterine involution rather than an indication of pathology. In agreement with these observations, our results showed predominance of *Firmicutes* during the first postpartum week, followed by increased relative abundance of *Fusobacteria* and *Bacteroidetes* by the ninth postpartum week in both treatment and control groups.

Although *Fusobacteria* and *Bacteroidetes* are often associated with postpartum uterine disease [8], several studies have reported their presence in cows without clinical signs of endometritis, suggesting that their presence alone does not indicate pathology [8,24]. Based on these data, all cows included in this study were clinically and reproductively healthy, and no signs of uterine disease were observed throughout the study, despite the presence of taxa commonly classified as uterine pathogens.

While causal relationships cannot be established, cows receiving slow-release grape pomace boluses exhibited distinct microbial profiles at nine weeks postpartum compared with control cows. Notably, the relative abundance of *Lactobacillus*-associated taxa was higher in supplemented cows, whereas control cows showed a stronger enrichment of *Fusobacteria* and *Actinobacteria*, including *T. pyogenes*. *Lactobacillus* spp. have been associated with microbial stability in the bovine reproductive tract [6]. These findings are consistent with previous studies demonstrating that polyphenol-rich diets can selectively promote lactic acid bacteria while suppressing opportunistic or pro-inflammatory taxa in the gastrointestinal tract [28,29]. However, evidence for similar mechanisms in the bovine uterus remains limited, and the present observations should therefore be interpreted as exploratory.

Cows receiving grape pomace supplementation exhibited a higher relative abundance of *Lactobacillus* spp. and a reduced presence of opportunistic fungal taxa, including *Candida* spp., compared with control animals. Similar microbiota-modulating effects of grape-derived polyphenols have been reported in previous studies, where polyphenol-rich diets promoted the growth of lactic acid bacteria and inhibited potentially pathogenic or opportunistic microorganisms [28,30,31].

*Lactobacillus* spp. have been commonly associated with microbial stability in the bovine reproductive tract, whereas an excessive presence of *Candida* spp. has been linked to microbial imbalance and inflammatory uterine conditions, including endometritis [31,32,33,34,35]. In the present study, the microbial patterns observed in supplemented cows were consistent with previously described antimicrobial and microbiota-modulating effects of plant-derived polyphenols, characterized by a more balanced microbial community structure.

However, as this study was designed for microbial profiling and relied on pooled samples, individual-level variation and causal relationships between dietary supplementation, microbial modulation, host immune responses, and reproductive performance could not be assessed. Therefore, the findings should be interpreted as descriptive and hypothesis-generating. Further studies incorporating larger sample sizes, individual-level sampling, and integrated clinical and reproductive data are required to confirm or refute these observations and to clarify their biological relevance.

## 5. Conclusions

This study showed that the uterine microbiota of beef cows changes over time during the postpartum period and is affected by dietary grape pomace supplementation. Differences in bacterial community composition were observed between the first and ninth weeks after calving. *Firmicutes* were more abundant in the early postpartum period, whereas *Fusobacteria* and *Bacteroidetes* increased later, especially in cows that did not receive supplementation. Cows supplemented with slow-release grape pomace boluses tended to show a lower relative abundance of bacteria commonly associated with uterine infections, such as *Fusobacterium necrophorum* and *Trueperella pyogenes*, and a more gradual change in microbial composition during uterine recovery. The fungal community was dominated by *Ascomycota* in all groups, but its abundance and species composition differed depending on postpartum stage and treatment. At nine weeks postpartum, control cows showed a higher proportion of opportunistic fungi, while supplemented cows had a more stable fungal community and lower prevalence of potentially pathogenic genera, including *Candida*. These results suggest a potential association between grape pomace supplementation and changes in both bacterial and fungal uterine microbiota during the postpartum period. Further research is needed to confirm these findings and to evaluate their relationship with reproductive performance.

## Data Availability

Data could be obtained from the corresponding author upon request. Sequences were deposited in the NCBI database through access number PRJNA1321294.
